# Overview of the structure and function of the dopamine transporter and its protein interactions

**DOI:** 10.3389/fphys.2023.1150355

**Published:** 2023-03-03

**Authors:** Binod Nepal, Sanjay Das, Maarten E. Reith, Sandhya Kortagere

**Affiliations:** ^1^ Department of Microbiology and Immunology, Drexel University College of Medicine, Philadelphia, PA, United States; ^2^ Department of Psychiatry, New York University School of Medicine, New York City, NY, United States

**Keywords:** α-synuclein, allosteric modulators, dopamine transporter, inward-open, oligomerization, outward-open, post translational modifications, tat

## Abstract

The dopamine transporter (DAT) plays an integral role in dopamine neurotransmission through the clearance of dopamine from the extracellular space. Dysregulation of DAT is central to the pathophysiology of numerous neuropsychiatric disorders and as such is an attractive therapeutic target. DAT belongs to the solute carrier family 6 (SLC6) class of Na^+^/Cl^−^ dependent transporters that move various cargo into neurons against their concentration gradient. This review focuses on DAT (SCL6A3 protein) while extending the narrative to the closely related transporters for serotonin and norepinephrine where needed for comparison or functional relevance. Cloning and site-directed mutagenesis experiments provided early structural knowledge of DAT but our contemporary understanding was achieved through a combination of crystallization of the related bacterial transporter LeuT, homology modeling, and subsequently the crystallization of *drosophila* DAT. These seminal findings enabled a better understanding of the conformational states involved in the transport of substrate, subsequently aiding state-specific drug design. Post-translational modifications to DAT such as phosphorylation, palmitoylation, ubiquitination also influence the plasma membrane localization and kinetics. Substrates and drugs can interact with multiple sites within DAT including the primary S1 and S2 sites involved in dopamine binding and novel allosteric sites. Major research has centered around the question what determines the substrate and inhibitor selectivity of DAT in comparison to serotonin and norepinephrine transporters. DAT has been implicated in many neurological disorders and may play a role in the pathology of HIV and Parkinson’s disease *via* direct physical interaction with HIV-1 Tat and α-synuclein proteins respectively.

## 1 Introduction

The solute carrier 6 (SLC6) family consists of a class of membrane proteins that utilize the Na^+^/Cl^−^ gradient to transport various substrates across the membrane (Pramod et al., 2013). The substrates include monoamines such as dopamine (DA), serotonin, norepinephrine and other neurotransmitters like GABA, amino acids or amino acid-like molecules such as glycine, creatine, and taurine. SLC6A3 is the dopamine transporter (DAT) involved in the release and reuptake of dopamine from the synapse and extrasynaptic space into neurons and is integral to dopaminergic neurotransmission (Amara and Pacholczyk, 1991). DAT is localized predominantly to DA neurons that form the mesolimbic, mesocortical, and mesostriatal pathways further supporting the role of DAT in dopaminergic neurotransmission (Ciliax et al., 1999). DAT is abundantly visualized using immunohistochemistry in cell bodies, axons and dendrites of DA neurons in the ventral tegmental area (VTA), substantia nigra (SNc) (Ciliax et al., 1995; Nirenberg et al., 1997a), and presynaptic terminals of the nucleus accumbens (NAc) (Xu and Chen, 2020). In the mesocortical pathway, DAT has been localized to motor, prefrontal, anterior cingulate, and visual cortex suggesting a major role in motivational and cognitive behaviors (Ciliax et al., 1999). Dysregulation of DAT is linked to several disorders including depression, bipolar disorder (BD), and attention-deficit/hyperactivity disorder (ADHD) (Reith et al., 2022). DAT is a major target for drugs of abuse including cocaine, amphetamine (AMPH), and methamphetamine (METH) as well as various medications such as methylphenidate and bupropion. In this review, we will summarize the structure, function, conformational dynamics, posttranslational modifications (PTMs), and interactions of DAT with ligands and other proteins such as Tat and α-synuclein.

## 2 Structure of DAT

**FIGURE 1:**
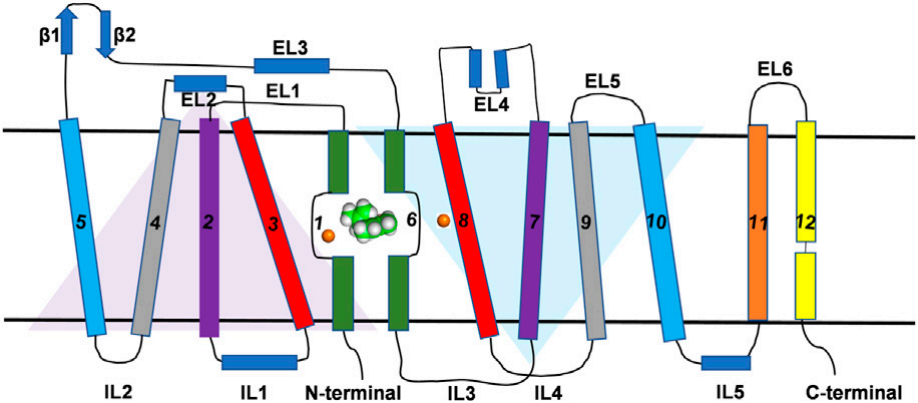
Topological representation of DAT. The TM helices, intracellular loops and extracellular loops are labeled. The orange spheres represent the Na^+^ ions. Green and white spherical model represents the substrate molecule. Two inverted triangles represent the two domains with an inverted axis of symmetry that contributes to substrate binding pocket.

All SLC6 transporters possess 12 transmembrane (TM) helices and share a high degree of sequence homology and three-dimensional structural architecture. The structural and conformational information of most members of SLC family including DAT was obtained from the bacterial leucine transporter (LeuT) which has high sequence similarity to the eukaryotic SLC6 transporters (Penmatsa and Gouaux, 2014; Grouleff et al., 2015). The crystal structure of LeuT was first resolved in 2005 and all early efforts at developing structure-function studies of DAT were inferred from homology models using LeuT as a template (Indarte et al., 2008). Recently the crystal structures of both transport-inactive and transport-active *drosophila melanogaster* DAT (dDAT) have been co-crystallized with a variety of ligands and these structures have provided more accurate insights into the structural models of human DAT (hDAT) (Penmatsa et al., 2013; Wang et al., 2015). Crystal structures of dDAT confirmed the architecture of hDAT is comprised of 12 TM helices interspersed with intracellular and extracellular loops (Figure 1). Similar to the structure of LeuT, TM one to five and 6–10 exhibit pseudo-two-fold symmetry along the plane of the membrane as represented by two triangles in Figure 1. The non-helical mid-regions of TM1 and TM6 along with TM3 and TM8 provide the binding site for the substrate and the ions while TM11 and TM12 occupy the peripheral position. Although the structure shows high-level similarity with LeuT, there are some specific structural and functional differences that make DAT specific for the transport of dopamine across the membrane. For example, a kink in the mid-region of TM12 (due to Pro572) in dDAT is different from LeuT. Additionally, cholesterol molecules occupy the grooves between TM5:TM7 and TM2:TM7 which have been demonstrated to influence the conformation of DAT during the transport cycle of the substrate (Hong and Amara, 2010; Zeppelin et al., 2018). While dDAT and hDAT have similar structural topology, caution is required to interpret structure-activity relationships of inhibitors that bind to hDAT because dDAT has a norepinephrine transporter-like inhibitor pharmacology (Pörzgen et al., 2001).

## 3 Conformational dynamics of DAT

**FIGURE 2:**
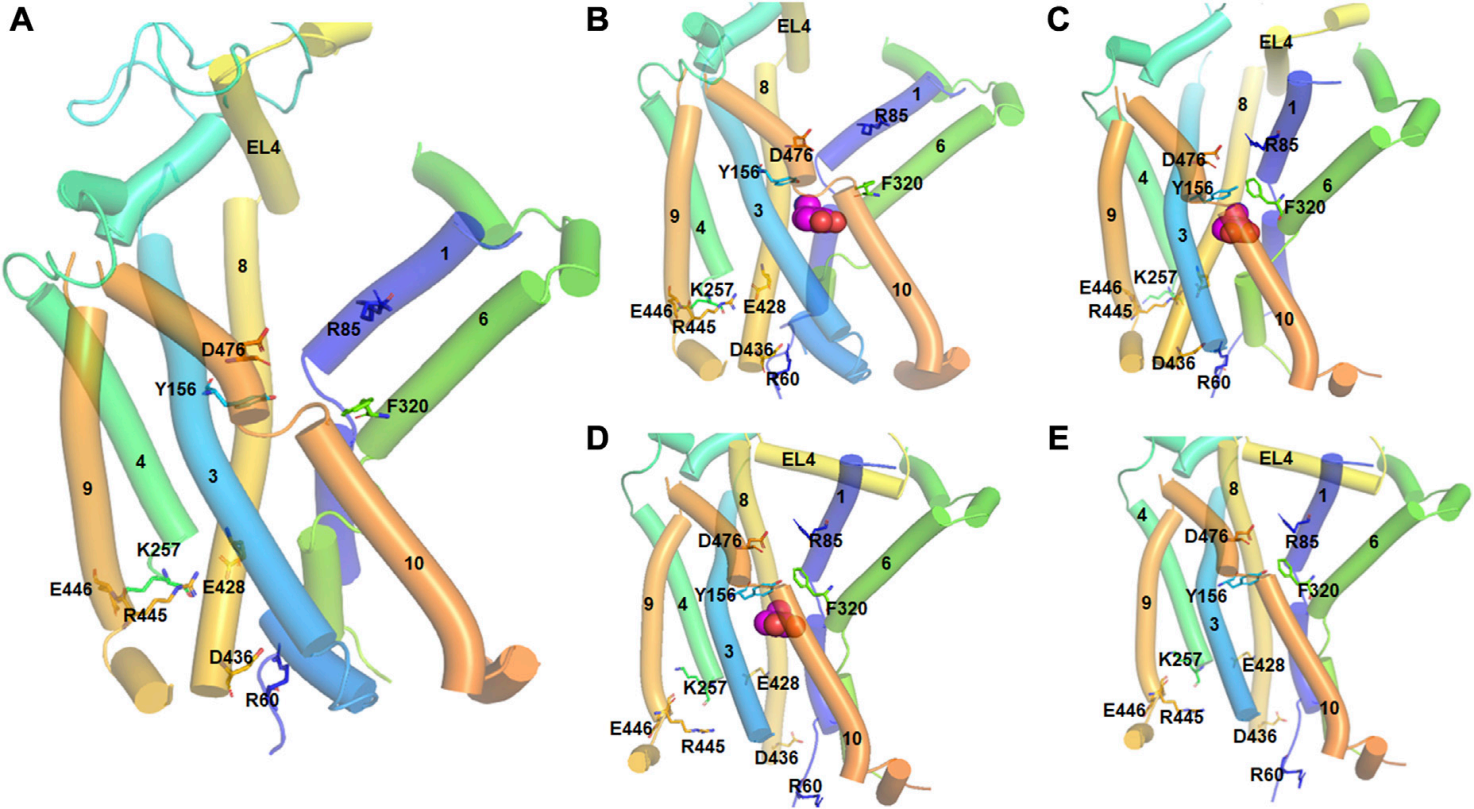
Different conformational states of hDAT during the transport cycle. The TM helices are numbered and represented by tubes. Important residues involved in conformational changes are shown in stick model and labeled. Substrate molecule (DA) is represented in spherical model. **(A)**. In the outward-open state (apo form), the salt bridge between D476 (TM10) and R85 (TM1) which acts as the first extracellular gate is interrupted. Similarly, the interaction between Y156 and F320 which function as the second extracellular gate is also broken. At the intracellular side, there are strong ionic interactions between E448-K257, R445-E428 and D436-R60 residues. Particularly, D436-R60 acts as the intracellular gate keeper. **(B)**. In the outward-open substrate bound state, a DA molecule binds at the orthostatic binding site. All the ionic interactions are the same as in the apo form. **(C)**. In the substrate-occluded state, the extracellular gates, D476-R85 and Y156 and F320 are closed. There is also movement of the EL4 loop to close the extracellular vestibule. **(D)**. In the inward-open state, the ionic interactions E448-K257, R445-E428 are interrupted, and the intracellular gate formed by D436-R60 is opened. **(E)**. In the inward-open state (apo from), DA is released into the intracellular sites and the cycle is reset.

The crystal structure of bacterial LeuT in various conformational states provided an excellent insight into the events of the transport cycle of DAT (Penmatsa and Gouaux, 2014). During the transport cycle, DAT attains three putative conformational states: outward-open, substrate-occluded, and inward-open states (Figure 2). In addition, intermediate states including the outward-occluded and inward-occluded states that can be inferred from the crystal structures of LeuT and SERT (MaryCheng and Bahar, 2015; Coleman et al., 2019; Gotfryd et al., 2020). The outward-open structure is the apo form of the protein and opens towards the extracellular site. After the substrates and ions enter through the opening and bind to their respective sites, extracellular loop 4 (EL4) locks the opening of the gate and the transporter shifts to the substrate occluded state. Subsequently, the transporter undergoes conformational changes resulting in the opening of the lumen to the intracellular side and giving it the aptly named inward-open state. The crystal structures of dDAT are only available in the outward-open conformational state (Penmatsa et al., 2013; Wang et al., 2015); however, the crystal structures of LeuT have provided additional insight into the structural changes associated with other transition states. Site-mutagenesis experiments and molecular dynamics (MD) simulations have revealed further details on the role of specific residues and the movement of specific TM domains within DAT (Loland et al., 2002; Loland et al., 2004; Kniazeff et al., 2008; MaryCheng and Bahar, 2015; Xu and Chen, 2022). In particular, the intracellular network between the N-terminus TM1, intracellular loop 1 (IL1), IL3 and TM8 consisting of ionic and cation-pi interactions is conserved among many members of the SLC6 family (Kniazeff et al., 2008). This network of interactions is believed to contribute to the opening and closing of the intracellular gate (Kniazeff et al., 2008). In hDAT, salt bridge between Arg60 and Asp436, and cation-pi interaction between Arg60 and Tyr335 have been identified to stabilize the outward-open conformation (Kniazeff et al., 2008; Kniazeff et al., 2008). Tyr335, located in IL3, largely alters the distribution between different conformational states in the transport cycle and modulates the effect of Zn^2+^ binding to hDAT (Loland et al., 2002) which is predicted to bind and stabilize the outward-open state (Loland et al., 1999; Stockner et al., 2013).

A study by Cheng et al. using all atom MD simulations demonstrated that TM5 and TM10 reorientations facilitate the transition from outward-open to inward-open states (MaryCheng and Bahar, 2015). This study also showed that the formation of a salt bridge between Arg445 and Glu428 is critical for the stability of the open state of the intracellular vestibule. Site-directed mutagenesis and structure-based analysis have revealed that this salt bridge enforces the inward-closed protein state, as R445 acts as a trigger for the inward-open state when the salt bridge is broken (Reith et al., 2018).

Recently, the importance of the N-terminal region in the conformational dynamics of hDAT was highlighted by MD simulations (Xu and Chen, 2022). This study demonstrates that when the N-terminal region interacts with the various intracellular loops and C-terminus, hDAT tends to attain an outward-open conformation. Conversely, when the N-terminal region mainly occupies the cytosolic region, an inward-open conformation of hDAT is observed.

A representative set of DAT substrates/inhibitors with their putative binding site and DAT’s preferred conformational state is indicated in Table 1. The conformational preferences were determined experimentally using either the cysteine accessibility experiments or by comparing binding affinity to specific mutants that shift the conformational state. For example, Tyr335Ala mutation shifts the equilibrium towards an inward-open state (Loland et al., 2002; Loland et al., 2004) while Trp84Leu and Asp313Asn mutation bias DAT conformation towards the outward-open state (Chen et al., 2001). Solvent accessibility measurements indicate Cys129 is highly solvent accessible in the outward-open state of DAT but not in the inward-open or occluded state (Loland et al., 2008). Similarly, the Ile159Cys mutant (in EL2 of DAT) provides better accessibility to bind to either R or S modafinil (Loland et al., 2012). Most DAT inhibitors have the propensity to bind either in the outward-open or the substrate-occluded states of DAT (Table 1). A few inhibitors such as JHW007 (benztropine analogue), SRI-31142, GBR12909, and ibogaine tend to stabilize the inward-open state of DAT and represent a type of atypical inhibitors (Reith et al., 2015; Newman et al., 2019). Computational docking studies have provided valuable insights into their binding mode and suggest that they may bind to either the S1 or the S2 site without disrupting the hydrogen bond gating between Asp79 and Tyr156 (Schmitt and Reith, 2011) which acts as an extracellular gate and preventing the transition of DAT to the outward-open state. In contrast, typical inhibitors like cocaine disrupt this extracellular gate thereby favoring an outward-open state of DAT.

**TABLE 1:** List of some DAT substrates/inhibitors with their preferential binding site and DAT’s conformational state.

Ligand	Site Preference	Configuration preference
Allotropacocaine		Closed/inward-facing Schmitt et al. (2013)
Amphetamine	S1 Wang et al. (2015)	Closed Cheng et al. (2015)
Benztropinamines	S1 Zou et al. (2017)	Induce inward-open Zou et al. (2017)
Benztropines analogs	S1, S2 Bisgaard et al. (2011)	Occluded Loland et al. (2008)
Bupropion		Closed/inward -facing Schmitt et al. (2013)
Cocaine and cocaine analogs	S1, S2 Beuming et al. (2008)	Outward-open Beuming et al. (2008)
Dopamine	Orthosteric Cheng et al. (2015)	Occluded Cheng et al. (2015)
GBR12909		Closed/inward facing Schmitt et al. (2013)
Ibogaine	S2 (Bulling et al., (2012)	Inward-open Bulling et al. (2012)
JHW007	S1 Bisgaard et al. (2011)	Closed/inward -facing Schmitt et al. (2013)
KM822	Allosteric Aggarwal et al. (2019)	Outward-open Aggarwal et al. (2019)
MDPV	S1 Steele et al. (2021)	Outward-open Steele et al. (2021)
Methamphetamine	S1 Wang et al. (2015)	Outward-open Wang et al. (2015)
Methylphenidate	S1 Foroughi et al. (2020)	Outward-open Schmitt and Reith, (2011)
MFZ 2–24	S1 Krout et al. (2017)	Outward-open Krout et al. (2017)
(±)-modafinil	S1 Schmitt and Reith, (2011)	Occluded Nikiforuk et al. (2017)
MRS7292	Allosteric Navratna et al. (2018)	Outward-open Navratna et al. (2018)
Nisoxetine	S1 Penmatsa et al. (2015)	Outward-open Penmatsa et al. (2015)
Nortriptyline	S1 Penmatsa et al. (2013)	Outward-open Penmatsa et al. (2013)
Orphenadrine	S1 Cheng et al. (2015)	Outward-open Cheng et al. (2015)
Reboxetine	S1 Penmatsa et al. (2015)	Outward-open Penmatsa et al. (2015)
Rimcazole		Closed/inward-facing Schmitt et al. (2013)
Rimcazole analogs		Occluded Loland et al. (2008)
RTI 82	S1 Dahal et al. (2014)	Outward-open Dahal et al. (2014)
SRI-31142	Allosteric Moerke et al. (2018)	Inward-facing Moerke et al. (2018)
Sydnocarb	Allosteric near EC Aggarwal et al. (2021)	Outward-open Aggarwal et al. (2021)
Tamoxifen	S2 site Mikelman et al. (2018)	Outward open Mikelman et al. (2018)
WIN 35, 428	S1 Beuming et al. (2008)	Outward-open Schmitt and Reith, (2011)

The binding of both substrate and Na^+^ was also shown to trigger conformational changes in DAT (Cheng et al., 2018; Nielsen et al., 2019). Shan *et al.* proposed an allosteric mechanism for the substrate transport in DAT (Shan et al., 2011). The binding of the second substrate molecule at the S2 site triggers the conformational changes at the S1 site which leads to the permeation of the water molecules at the S1 site and facilitates the inward-open conformation. The hinge regions in TM1 and TM6 enable these large conformational transitions. Cholesterol also plays an important role in stabilizing the outward-open conformation (Jones et al., 2012). In addition to these two sites, other allosteric sites that are conformation-specific have been identified using MD simulations and site directed mutagenesis/transporter truncation methods (Aggarwal et al., 2019). Small molecule allosteric modulators such as KM822 bind to these sites and allosterically modulate the binding of cocaine while restoring the substrate function (Mortensen and Kortagere, 2015; Aggarwal et al., 2019).

In a recent study, we mapped familial mutations associated with several neurological and neuropsychiatric disorders in DAT (Reith et al., 2022). Some of these mutations are located at domains of DAT that are involved in promoting conformational changes when DAT undergoes transitions during the substrate transport cycle. For example, Thr356 in hDAT facilitates a change in hDAT to the outward-open conformation upon substrate binding and promotes the substrate efflux, however, the mutation Thr356Met associated with autism spectrum disorder blocks conformational changes in hDAT leading to effects on transport cycle (Hamilton et al., 2013). Similarly, Thr62Asp-mutated hDAT is locked in an inward-open conformation. The inward-open to outward-open conformational dynamics were affected by a Tyr470His mutation in hDAT (Midde et al., 2013). Leu368Gln and Pro395Leu mutations in hDAT are found to be associated with infantile parkinsonism-dystonia presumably due to the alteration in the conformational dynamics of its transport cycle (Kurian et al., 2009). Ala559Val mutation in hDAT is linked to ADHD and this mutant DAT showed elevated levels of DA efflux at the depolarization potential (Mazei-Robison et al., 2008). Ile312Phe and Asp421Asn mutations in hDAT are associated with adult parkinsonism and ADHD (Hansen et al., 2014). Asp421Asn mutation reduced sodium binding and is hypothesized to favor the inward-open conformation of hDAT (Hansen et al., 2014).

## 4 Post-translational modification of DAT

**FIGURE 3:**
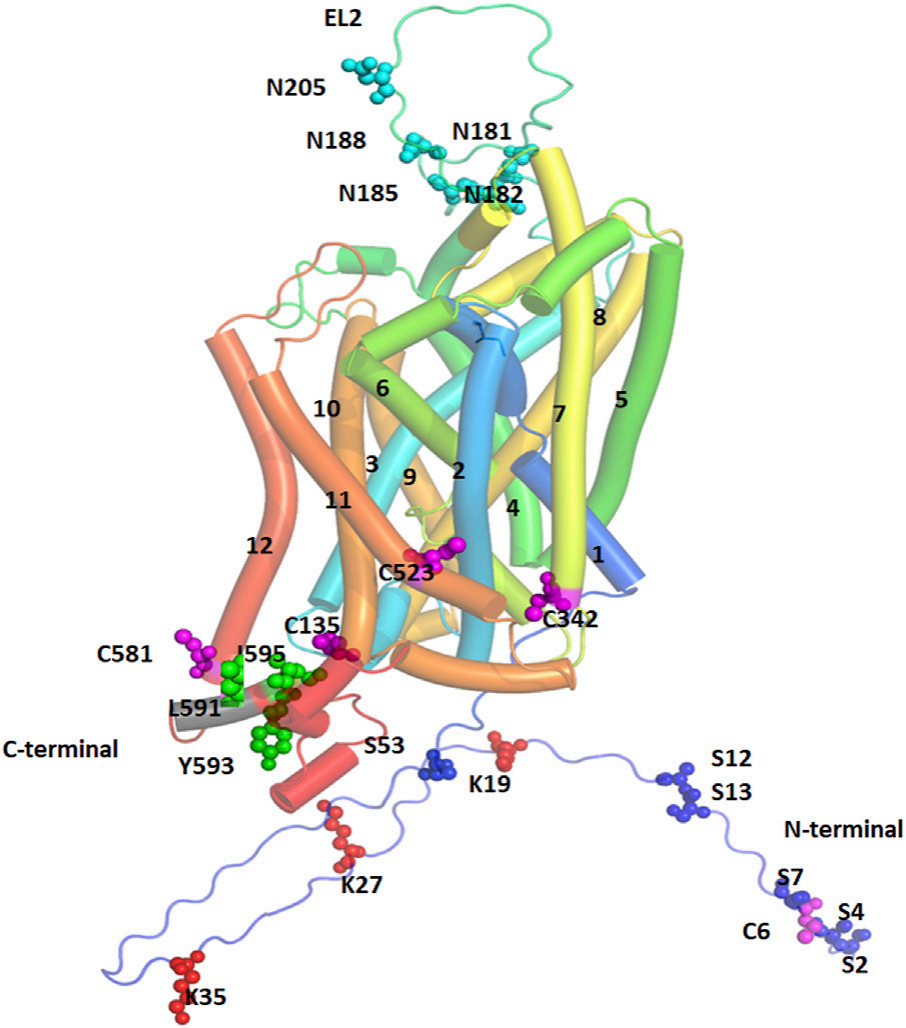
Post-Translational Modification (PTM) sites for hDAT are depicted. TM helices are labeled, and color coded. The sites of PTM are shown in spherical models and are color coded. Magenta spheres represent sites for palmitoylation, red spheres represent ubiquitination sites, cyan spheres represent sites for glycosylation, and green spheres represent the site for PKC-mediated endocytosis.

Synthesis of DAT protein occurs in the soma of SNc and VTA DA neurons. It is then processed and trafficked through the axonal processes projecting into the dorsal striatum and NAc terminals of DA neurons (Nirenberg et al., 1997b; Nirenberg et al., 1997a). Trafficking of DAT is tightly regulated by several posttranslational modifications (PTMs) including phosphorylation, palmitoylation, ubiquitination, and glycosylation which ultimately determine the localization of DAT to the cytoplasm or plasma membrane (Li et al., 2004; Sorkina et al., 2006; Gorentla et al., 2009; Vina-Vilaseca et al., 2011; Moritz et al., 2015) and regulate function by altering membrane kinetics (Patel et al., 1994; Li et al., 2004). Other regulatory mechanisms of DAT include nitrosylation and oxidation (Berman et al., 1996; Fleckenstein et al., 1997; Volz and Schenk, 2004). The sites for PTM are located on the N-terminal domain (NTD) or intracellular loop (IL) 1 and the C-terminal cytoplasmic domain (CTD) and the extracellular segments between the third and fourth transmembrane domains (EL2) (Figure 3).

### 4.1 Phosphorylation

Several sites of phosphorylation have been predicted for DAT that are distributed on its NTD and some of these have been validated experimentally including serine residues 2, 4, 7, 12 and 13 (Foster et al., 2002; Granas et al., 2003; Cervinski et al., 2005; Karam and Javitch, 2018), and Thr53 (Foster et al., 2012). Several serine and threonine residues in the NTD may be phosphorylated by protein kinase C (PKC) and protein kinase A (PKA), protein kinase G, and Ca^2+^/Calmodulin protein kinase II, ERK1, JNK, and P38 kinases (Giambalvo, 1992; Batchelor and Schenk, 1998; Giambalvo, 2003; Lin et al., 2003; Gorentla et al., 2009). Deletion of the first 22 residues of the NTD or mutations of the 5 NTD serine residues to alanine lead to loss of phosphorylation by PKC in cultured cells suggesting that transporter phosphorylation is almost entirely limited to the NTD (Granas et al., 2003). Proline-directed phosphorylation at Thr53 by extracellular kinase1/2 (ERK1/2) increases the membrane localization and activity of DAT (Vaughan and Foster, 2013; Foster and Vaughan, 2017). The point mutation of this residue decreases the Vmax of DAT, potentially important for reverse transport when induced by amphetamine (AMPH) (Gorentla et al., 2009; Foster et al., 2012).

Phosphorylation at Thr53 (pDAT) is implicated in many functions including sleep, stress, and cocaine effects. Sleep/wake state irrespective of light/dark phase is reported to influence DA uptake *via* pDAT (Alonso et al., 2021). Phosphorylation of DAT at Thr53 increases plasma membrane localization which enhances the rate of DA uptake (Schmitt and Reith, 2010; Foster et al., 2012; Calipari et al., 2017; Challasivakanaka et al., 2017). Further, phosphorylation of DAT at Thr53 also contributes to enhanced DAT sensitivity to cocaine and its analogs (Foster et al., 2012; Vaughan and Foster, 2013; Challasivakanaka et al., 2017). In these studies, pDAT levels correlated with cocaine and opioid induced increases in DAT kinetics (Alonso et al., 2021; Samson et al., 2022). However, PKC-mediated phosphorylation was found to reduce the Vmax of DAT and enhanced clathrin-mediated DAT internalization. Further, treatment with PKC inhibitors blocked the internalization of DAT (Daniels and Amara, 1999; Melikian and Buckley, 1999; Sorkina et al., 2005; Foster et al., 2008; Sorkina et al., 2013) (Foster et al., 2002). Membrane DAT abundance was found to be reduced by phorbol ester through PKC-mediated phosphorylation (Copeland et al., 1996; Huff et al., 1997; Vaughan et al., 1997; Zhu et al., 1997; Daniels and Amara, 1999; Melikian and Buckley, 1999; Karam and Javitch, 2018), or *via* diacylglycerol analogs (Vaughan et al., 1997), or by GPCR agonists mediated Gq activation (Granas et al., 2003). The transporter is dephosphorylated by protein phosphatase (PPI or PP2A), and metabolic labeling experiments using P^32^ labeling confirmed that when protein phosphatases are inhibited by okadaic acid, an increase in P^32^ labeling occurs, suggesting a steady state reduction in phosphorylated DAT (Yang et al., 2019) (Foster and Vaughan, 2017). Although the role of NTD serine phosphorylation remains unclear, it is assumed to be involved in protein-protein interactions, regulating constitutive DAT turnover, and altering substrate transport during stimulation exposures (Khoshbouei et al., 2004; Eriksen et al., 2010; Bu et al., 2021). PKC interaction at the CTD may be involved in DAT internalization by clathrin mediated endocytosis and the required residues at the CTD are Leu591, Tyr593 and Ile595 (Boudanova et al., 2008), whereas the segment of residues from Phe587 to Leu591 is implicated in DAT downregulation (Holton et al., 2005). Further, it was found that the removal of consensus sites for PKC-mediated phosphorylation failed to create any alteration in the transportation and the internalization activity of the transporter (Holton et al., 2005; Boudanova et al., 2008). Increasing evidence suggests the dysfunctional regulation of DAT is associated with disease by creating hypo or hyper DA’ergic conditions. DA’ergic disorders are mostly associated with genetic polymorphism which alters the phosphorylation of DAT (Hahn and Blakely, 2007). ADHD and BD are two examples wherein mutation of Ala559Val (extracellular end of TM12) (Mazei-Robison et al., 2008) and Arg615Cys (CTD CamKII binding site) have been identified as causal for the alterations in DA efflux due to transporter hyperphosphorylation (Sakrikar et al., 2012). Val328Ala is another polymorphism in the transporter found in the patients of Tourette’s syndrome where enhanced trafficking-independent downregulation occurs due to hyperdopaminergia by PKC domain phosphorylation (Mazei-Robison et al., 2005). For hypodopaminergic conditions associated with DAT phosphorylation several genetic disorders like Angelman Syndrome have been linked and a decrease in CAMKII activity leading to a decrease in DA efflux has been noted (Mabb et al., 2011; Dichter et al., 2012; Steinkellner et al., 2012).

### 4.2 Palmitoylation

DAT is palmitoylated (16-carbon fatty acid chain) *via* thioester bonding mainly at Cys580 in the membrane CTD helical region (Rastedt et al., 2017). Catalysis of palmitoylation is driven by palmitoyl acetyl transferase which is also known as DHHC derived from the short peptide composed of Asp-His-His-Cys in humans (Bolland et al., 2019). DAT is depalmitoylated by acyl protein thioesterase, a superfamily of serine hydrolases and palmitoyl protein thioesterase is associated with lysosomal degradation of DAT (Fukata and Fukata, 2010). Although not belonging to these families, another member of α/β hydrolase domain containing serine hydrolase was found to depalmitoylate neuronal Post Synaptic Density 95 protein suggesting the presence of other undiscovered proteins regulating DAT palmitoylation (Yokoi et al., 2016). Among the five possible conserved Cys residues in rodents (r) and human (h), each (rDAT: 6, 135, 341, 522, 580; hDAT: 6, 135, 342, 523 and 581) are exposed for palmitoylation, and mutational studies indicate 60% reduction in incorporation at the Cys580 (rDAT) site (Rastedt et al., 2017). Furthermore, Cys581 palmitoylation of hDAT leads to formation of stable and energetically favorable hDAT dimers with functional effects on DA uptake (Zeppelin et al., 2021). Various studies found that DAT palmitoylation increases the DA uptake Vmax and reduces the likelihood of degradation whereas depalmitoylation enhances DA efflux, decreases DA uptake Vmax, and increases the occurrence of lysosomal-associated degradation similar to the effects of phosphorylation (Khoshbouei et al., 2004; Moritz et al., 2015; Wang C. et al., 2016). Activation of PKC enhances Ser7 phosphorylation which consequently reduces DAT palmitoylation, and either treatment with PKC inhibitors or Ser7Ala mutation elevates DAT palmitoylation (Vaughan et al., 1997; Foster and Vaughan, 2011; Davda et al., 2013; Moritz et al., 2015). Impairment of DAT palmitoylation may lead to several psychiatric and neurological diseases as indicated by single nucleotide polymorphisms identified in ADHD and BD (Mazei-Robison et al., 2005). Reduction of palmitoylation was linked to alteration in phosphorylation leading to increase in DA efflux and hyperdopaminergia (Khoshbouei et al., 2004; Wang Q. et al., 2016). Thus, DAT phosphorylation and palmitoylation are reciprocally regulated and alteration in palmitoylation posits DA imbalance (Moritz et al., 2015).

### 4.3 Ubiquitination

Ubiquitination of DAT occurs in the NTD on lysine residues in a PKC-dependent manner (Miranda et al., 2005). Mutation of three lysine residues (Lys19, 27 and 35) in the NTD of DAT lead to loss of PKC-dependent ubiquitination and endocytosis of DAT (Miranda et al., 2007). In temporary ubiquitination, DAT is recycled back to the membrane *via* the constitutive recycling pathway whereas permanent ubiquitination leads to DAT degradation *via* the late endocytic lysosomal degradation pathway (Daniels and Amara, 1999; Miranda et al., 2005; Staub and Rotin, 2006; Miranda et al., 2007). Ubiquitin ligases which degrade DAT *via* the lysosomal degradation pathway include neural precursor cells developmental downregulated protein four to two or NEDD4-2 (Sorkina et al., 2006) and Parkin (Jiang et al., 2004).

Small ubiquitin like modifier1 (SUMO1) and Ubc9 SUMO were reported to increase DAT levels in the plasma membrane by inducing DAT SUMOylation and reducing the possibility of DAT ubiquitination and degradation (Cartier et al., 2019). Hence, SUMOylation plays a crucial and novel role in regulating DAT proteostasis, DA uptake, and DA signaling in neurons. SUMOylation is a potential therapeutic target to increase DAT activity and DA clearance in physiological and pathological states without the use of stimulants. Numerous neurological disorders including depression, ADHD, PD, and BD are associated with functional alteration of DAT levels caused by these PTMs (Vaughan and Foster, 2013). Mutation of Parkin related to DAT ubiquitination causes PD due to loss of regulation of DAT recycling and failure to ubiquitylate misfolded transporter (Jiang et al., 2004).

### 4.4 Glycosylation

DAT glycosylation has been deemed necessary for the localization of DAT to the plasma membrane and to increase DA transport rate and Vmax (Patel et al., 1994; Li et al., 2004). The asparagine residue (Asn181,188, 205) on the EL2 loop between TM 2 and 3 is the target for glycosylation of DAT and mutation or removal of those sites results in reduction or blocking of DAT glycosylation (Vaughan and Kuhar, 1996; Torres et al., 2003; Li et al., 2004). Partially or non-glycosylated DATs are preferentially endocytosed compared to fully glycosylated DATs (Torres et al., 2003; Li et al., 2004) and N-glycosylation of DAT may facilitate its oligomerization in relation to its intracellular trafficking (Torres et al., 2003). Furthermore, glycosylation increases DAT’s susceptibility to bind to various drugs such as cocaine (Li et al., 2004) and reduced glycosylation of DAT in the human striatum and midbrain leads to increased PD susceptibility (Afonso-Oramas et al., 2009). These studies validate DAT as a therapeutic target and provide avenues for pharmacological manipulation of DAT to reduce disease severity.

### 4.5 Nitrosylation and oxidation

Nitric oxide-mediated DAT nitrosylation was found to contribute to DAT regulation. Nitrosylation occurs on the Cys residues 135 and 342 (Pogun et al., 1994; Zahniser and Doolen, 2001). L-Arg, a substrate of nitric oxide synthase (NOS) was found to enhance DAT activity by either increasing the Vmax, or decreasing Km of DA uptake (Chaparro-Huerta et al., 1997; Volz and Schenk, 2004). L-Arg was further found to be effective by protecting against sulfhydryl agents like N-ethylmaleimide (Volz and Schenk, 2004). These results suggest that L-Arg is crucial in regulating DAT activity in rat striatum through nitric oxide (Volz and Schenk, 2004). Nitric oxide was further reported to increase (Lin et al., 1995; Chaparro-Huerta et al., 1997) or decrease (Lonart and Johnson, 1994; Pogun et al., 1994) DAT activity in rat striatum or inhibit DA uptake without accelerating reversed transport of substrates (Cao and Reith, 2002). Cocaine and DA were found to block nitrosylation by inhibiting the interaction of nitric oxide with DAT at Cys135 and Cys342 due to alteration in DAT structure, however Cys90 in EL2 could be a target for nitrosylation due to its increased accessibility in the outward open conformation (Meiergerd and Schenk, 1994a; Ferrer and Javitch, 1998; Reith et al., 2001).

In addition to nitrosylation, reactive oxygen species (ROS) were also found to decrease DAT activity (Berman et al., 1996; Fleckenstein et al., 1997). ROS-mediated oxidative stress during pathological conditions including ischemia and neurodegenerative disease decreases DAT activity. It is likely that DAT may be regulated by ROS in the form of DA quinonens (Berman et al., 1996). Lipid peroxidation in striatal slices was found to suppress DAT activity (Page et al., 1998) and peroxynitrile was found to reduce DAT activity by modulation of Cys342 in IL3 (Park et al., 2002). H_2_O_2_ produced either by physiological stimulation (Sundaresan et al., 1995; Bae et al., 1997) or pathological insult like ischemia (Hyslop et al., 1995) was further found to be potential detrimental factors for DAT activity (Huang et al., 2003).

## 5 DAT oligomerization

Evidence for DAT oligomerization was deduced using a radiation inactivation technique that identified the tetrameric form of DAT in canine striatal membranes (Milner et al., 1994). Oligomerization is one of the key characteristics of SLC6 transporters, playing a critical role in their physiological and functional activity (Jayaraman et al., 2021). Several pieces of evidence suggest oligomerization is essential for the transport of DAT from the endoplasmic reticulum (ER) to the plasma membrane during synthesis (Sorkina et al., 2003; Ingram et al., 2021). The presence of oligomeric structures in the endosome implies oligomerization has roles in DAT endocytosis and recycling (Sorkina et al., 2003). Additionally, DAT oligomers are found in the plasma membrane as dimers, tetramers, and other higher order molecular structures (Hastrup et al., 2001; Zhen and Reith, 2018) while cysteine crosslinking experiments have detected dimer, trimer, and tetramer plasma membrane forms (Hastrup et al., 2001; Hastrup et al., 2003; Sorkina et al., 2018). The oligomerization of the various SLC6 transporters can be modulated by lipid composition (Anderluh et al., 2017) but in contrast to other SLC transporters, perturbation of cholesterol and phosphatidylinositol 4,5-bisphosphate (PIP2) do not have an intrinsic impact on hDAT oligomerization as revealed by single-molecule brightness analysis (Das et al., 2019). Single molecule microscopy also unveiled that in the ER membrane (which is devoid of PIP2) and the plasma membrane (where PIP2 is present), mixtures of monomers up to pentamers occurred for hSERT (Anderluh et al., 2017) whereas monomers and dimers were observed for hDAT regardless of PIP2 presence (Anderluh et al., 2017). However, this does not discount the role of PIP2 in SLC6 oligomer dynamics as at ER, hSERT oligomer subunits are continuously exchanged, whereas neither hDAT nor hSERT exchange in the plasma membrane even over the course of minutes (Hastrup et al., 2001; Anderluh et al., 2017). It has been concluded that PIP2 is instrumental in determining the oligomeric state of hSERT and likely also hDAT on the plasma membrane (Hastrup et al., 2001; Zhen et al., 2015). Finally, PIP2 can modulate hSERT and hDAT function (uptake, efflux, current) through precise binding interactions (Buchmayer et al., 2013; Hamilton et al., 2014; Belovich et al., 2021); however, it is not known whether these PIP2 binding sites (core protein of hSERT and hDAT, and N-terminal of hDAT) play a direct role in the quaternary arrangements.

One of the critical questions to answer is the effect of hDAT oligomerization on function. A study by Zhen et al. using copper phenanthroline (CuP) cross-linked hDAT indicates dimerization decreases the uptake activity by 25% compared to the individual protomers (Zhen and Reith, 2018). This is consistent with previous reports on other monoamine transporters SERT and NET suggesting that only one protomer in the dimer is active at a time and the activity of the one monomer could antagonize the activity of the other (Kocabas et al., 2003). Besides the DA uptake activity, oligomerization of DAT also alters the binding affinity of some inhibitors. For example, the binding affinity of the cocaine analog 2β-carbomethoxy-3β-[4-fluorophenyl]-tropane (CFT) was reduced by almost five fold on hDAT homo-dimer cross-linked with CuP in comparison to the monomeric state (Zhen and Reith, 2018). Similarly, amphetamine-stimulated DA efflux was also decreased in the dimeric form compared to the monomeric form in the same study. However, the results are more complicated with DAT hetero-oligomers when the two protomers have differential inhibitor binding affinities. For instance, a study with co-expressed DAT constructs with varying binding affinities for CFT revealed that the binding affinity in hetero-oligomers can increase or decrease in comparison to singly expressed DAT constructs where such oligomers could not be detected (Zhen et al., 2015). This indicates that DAT protomers within the oligomers do not function independently, rather a significant cooperative effect occurs among the protomers. An earlier observation implicated oligomerization of SLC6 transporters in linking drug substrate uptake with dopamine efflux (Seidel et al., 2005) and one might consider substrate transport by this class of transporters as being asymmetric depending of oligomerization (Jayaraman et al., 2021).

It is important to consider the structural features of the oligomerization interface on DAT function; however, no resolved oligomeric state crystal structures of DAT or other monoamine SLC6 family members exist to date. The dimeric crystal structure of LeuT shows TM9 and TM12 form the oligomeric interface with EL2 and EL6 loops coordinating extracellularly (Krishnamurthy and Gouaux, 2012); though, due to the presence of a kink in DAT TM12 (Pro572), the DAT oligomerization interface may differ (Penmatsa et al., 2013). Cystine crosslinking, site-directed mutagenesis, and MD simulations have provided some insights but a complete picture of the structural basis of oligomerization in DAT is still not available. Since TM11 and TM12 are surface exposed, it is expected that these helices may reside at the oligomeric interface. Indeed, biochemical studies have suggested that TM11 and TM12 contribute to the oligomeric interfaces in hSERT (Just et al., 2004). Cysteine crosslinking suggests that Cys243 in TM4 and Cys306 at the extracellular end of TM6 contribute to the oligomeric interface of hDAT dimers and higher order oligomers (Hastrup et al., 2001; Hastrup et al., 2003). Site-directed mutagenesis indicates that leucine zipper-like motifs present in TM2 may play a critical role in DAT in oligomerization (Torres et al., 2003). Similarly, truncation of the C-terminal of the hDAT suggests that the intracellular C-terminus is essential for the trafficking of DAT to the plasma membrane but does not play a significant role in oligomerization (Torres et al., 2003).

Palmitoylation on TM12 has a favorable impact on oligomerization but the functional consequences on DAT are unclear (Zeppelin et al., 2021). Many attempts to understand the conformational dynamics of DAT oligomers and to predict distinct oligomeric interfaces have been made using MD simulations (Gur et al., 2017; Jayaraman et al., 2018; Zeppelin et al., 2021). Coarse-grained MD simulations of hDAT show that TM9, TM11, and/or TM12 provide the dimeric interface for oligomerization (Zeppelin et al., 2021). Unrestrained and unbiased coarse-grained MD simulation show that hDAT can form stable dimers through 6 different interfaces that include both symmetric and asymmetric dimers (Jayaraman et al., 2018). Additionally, this simulation indicates that TM4, TM9, TM5, TM11, and extracellular and intracellular terminal ends of TM3, TM8, TM12 contribute to the dimer interface. Unfortunately, this study was unable to identify the most relevant dimer conformation that could explain DAT function (Jayaraman et al., 2018). Gur *et al.* carried out MD simulations of dimers of hDAT in both inward-open and outward-open conformations with dimers containing TM2, TM6, and TM11 at the oligomeric interface with Cys306 at TM6 facing each other (Gur et al., 2017). The authors predicted that dimerization facilitates the transition between inward-open to outward-open conformation enabling higher substrate transport efficiency in DAT compared to the monomeric forms; however, experimental evidence suggests dimerization decreases transport efficiency by nearly 25% for hDAT (Zhen and Reith, 2018). The effect of the small molecule inhibitor AIM-100 on inducing trimerization of DAT was studied by combined site-directed mutagenesis and MD simulation (Sorkina et al., 2021). The results show that DAT trimerization is favored by inward-open conformation, involves the hydrophobic residues in the TM4 and TM9, and inhibitors like AIM-100 facilitate the transition from outward-open to inward-open conformation (Sorkina et al., 2021).

Oligomerization of DAT on the cell surface can also be modulated by substrates and various inhibitors. Methamphetamine and AIM-100 are reported to induce higher order DAT oligomers in turn decreasing the concentration of DAT on the plasma membrane (Baucum et al., 2004; Cheng et al., 2019). AIM-100 can promote dynamin-independent DAT endocytosis presumably due to DAT oligomerization (Sorkina et al., 2021). The induction of oligomerization by methamphetamine is also observed in an *in vivo* study in rats (Baucum et al., 2004). However, not all stimulants can promote oligomerization of DAT as cross-linking with copper sulfate phenanthroline shows that both dopamine and amphetamine decrease DAT oligomerization (Chen and Reith, 2008; Li et al., 2010). In agreement with this result, amphetamine was also found to dissociate DAT oligomers induced by cocaine treatment (Siciliano et al., 2018). The above observations suggest that inhibition of DAT can regulate its oligomerization state and further influence its surface expression and function.

## 6 DAT ligand interaction sites S1, S2, and allosteric sites

**FIGURE 4:**
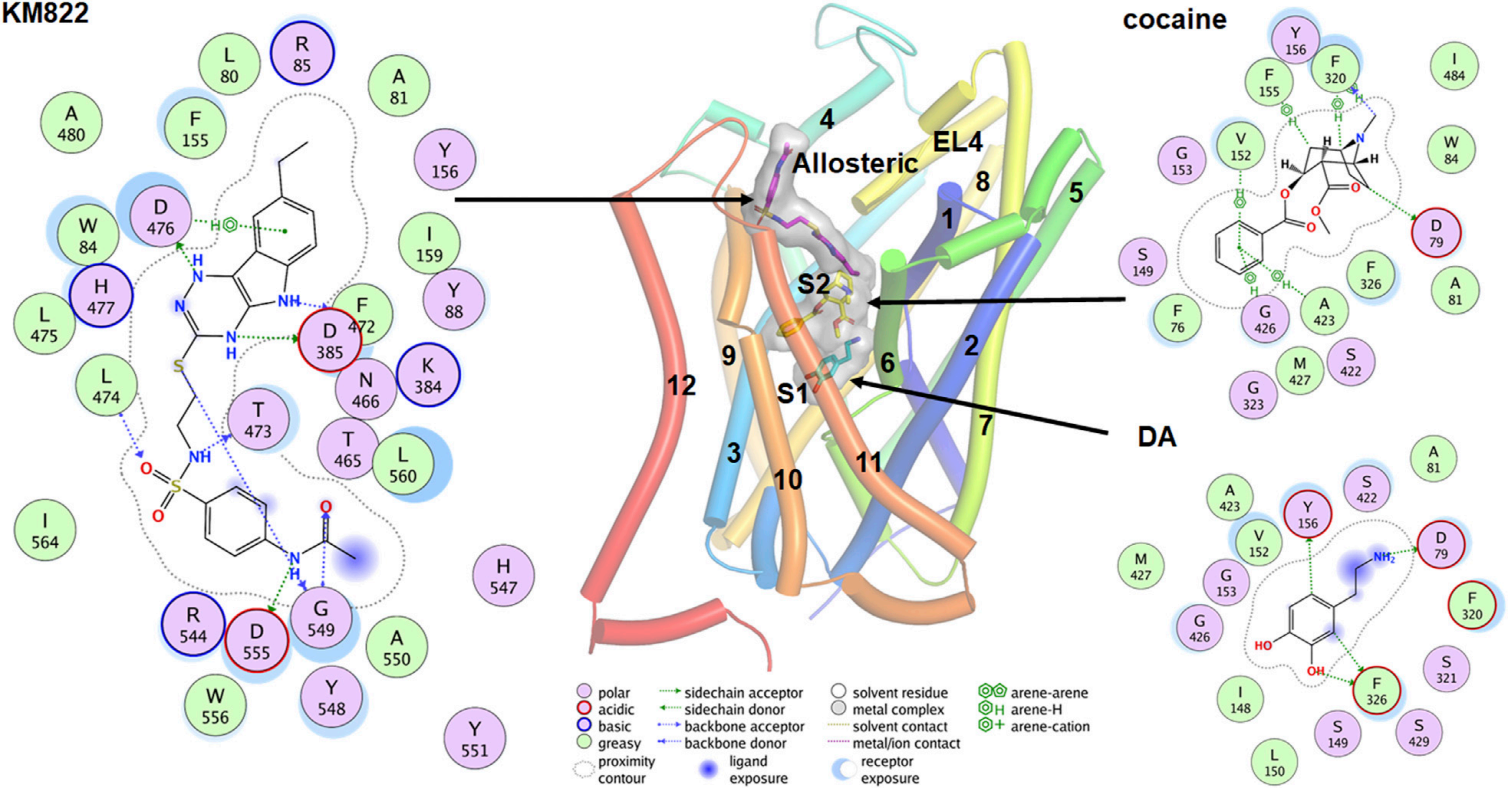
Binding poses for the DA, cocaine and KM822 molecules at the orthosteric (S1), S2, and allosteric site respectively. The ligand molecules are shown by the stick model with a transparent surface. The 2D ligand interactions map is also shown for the ligands.

DAT binds to several classes of ligands besides dopamine including stimulants such as cocaine and amphetamine, antidepressants like imipramine, and the allosteric modulator KM822 (Table 1) (Tatsumi et al., 1997; Saunders et al., 2000; Schmitt et al., 2013; Reith et al., 2015; Nikiforuk et al., 2017; Aggarwal et al., 2019). These ligands bind at distinct sites and hence differentially affect the activity of DAT. Early evidence for the binding of these various ligands to DAT came from site-directed mutagenesis and kinetic experiments (McElvain and Schenk, 1992; Reith and Selmeci, 1992; Meiergerd and Schenk, 1994b; Wayment et al., 1998; Lin et al., 1999; Wayment et al., 1999). These studies confirmed the effects of inhibitors and substrates on DAT function but not on their binding pose at the transporter leading to inconclusive reports on the binding of cocaine and its mechanism of action (Wayment et al., 1998; Loland et al., 2004; Chen et al., 2005; Sen et al., 2005). The crystal structures of LeuT (Yamashita et al., 2005) and dDAT (Penmatsa et al., 2013; Wang et al., 2015) with validation from site-directed mutagenesis have provided reliable results (Singh et al., 2007; Zhou et al., 2007). Accordingly, the orthosteric binding pocket, also called the S1 site, is formed by the non-helical mid-regions of TM1 and TM6 along with the mid-regions of TM3 and TM8 which binds dopamine (Figure 4). Residue Asp79 in TM1 forms a salt bridge with the amine group of dopamine, while Tyr156 in TM3 forms π-π interactions with the catechol ring. Further, Val152, Phe76, and Phe326 form the hydrophobic-aromatic cluster interactions with the catechol ring. The binding site for cocaine and its tropane analogs has been debated with some reports suggesting binding occurs at the orthosteric site while other reports demonstrating that the cocaine binds at a distinct site with shared residues from the S1 site (Figure 4) (Beuming et al., 2008; Huang et al., 2009; Wang et al., 2015). Based on the co-crystallization of various inhibitors with LeuT, a second site referred to as the S2 site was identified approximately 11Å above the substrate binding site in a vestibule open toward the extracellular side (Shi et al., 2008; Nyola et al., 2010; Khelashvili et al., 2013). This site has been shown to bind several non-competitive and allosteric inhibitors of DAT (Aggarwal et al., 2021).

Co-crystallization of DAT with the relevant ligand provides more direct evidence for the possible binding sites. Co-crystallization of the antidepressant nortriptyline with inactive *drosophila* DAT (dDAT) indicated that nortriptyline binds at the S1 binding site (Penmatsa et al., 2013). Similarly, co-crystallization of various ligand including dopamine, D-amphetamine, methamphetamine, cocaine, and cocaine analogs with active dDAT indicates all ligands bind at the same orthosteric binding site (Wang et al., 2015). Conformational changes in dDAT accommodate the different-size ligands at the same site (Wang et al., 2015). An MD simulation predicted that cocaine binds near the extracellular end of the substrate-entry tunnel bounded by TMs 1, 3, 6, 8, and 10 (site S2). In this conformation, Tyr88, Ile390, Phe391, and Phe472 interact with the charged head group and Leu80, Phe155, Tyr156, Ile159, and Phe330 provide a hydrophobic cavity for the benzoyl ester group of cocaine (Huang et al., 2009). A steered molecular dynamics (SMD) simulation predicted that substrate at site S2 causes a substantial allosteric effect at site S1 (Shan et al., 2011), shifting the center of mass of the substrate at the S1 site by as much as 1Å. Cheng *et al.* conducted combined docking and MD simulations for the binding of dopamine, amphetamine, orphenadrine, and cocaine in the hDAT model. Although their docking result showed that all ligands tend to bind near the vicinity of the S1, MD simulations showed that larger-size ligands are also stabilized at the S2 site (Cheng et al., 2015). A more recent docking and MD simulation study demonstrates that in addition to the S1, cocaine can also bind in another new allosteric site formed by TMs 3, 9, 10, and 12 (Xu and Chen, 2020). The authors claim this allosteric site has a higher affinity for cocaine than site S1, and that the binding of cocaine in this site induces profound conformational changes inhibiting the binding of dopamine at site S1 (Xu and Chen, 2020).

Although there is a substantial discrepancy in the possible binding sites for different inhibitors, it is likely that substrates bind to the S2 site and slowly translocate to the S1 site which is accompanied by some conformational changes. Related studies with bivalent substrate ligands by Schmitt et al. (2010) showed increasing DAT affinity. In this study a series of bivalent phenethylamines with the two phenylalkylamine moieties separated by an aliphatic spacer of increasing length were investigated, with one compound with an 8-carbon linker displaying an 82-fold gain in affinity. Computational modeling confirmed that the two dopamine-like pharmacophoric “heads” of this bivalent compound simultaneously occupied S1 and S2. Anderson et al*.* (2016) also provided evidence that both S1 and S2 can bind substrate with high affinity. The authors synthesized combinations of bivalent substrates (two substrate molecules connected by a flexible linker) which can bind to the sites S1 and S2 simultaneously. These experiments show that bivalent substrates bind with significantly higher affinity compared to their corresponding monovalent substrates and suggest that both binding sites are relevant for binding the substrate molecules.

Ligands which bind at sites other than S1 and S2 have great pharmacological potential. For example, ligands which selectively bind to the allosteric sites and inhibit the binding of cocaine without modulating DAT uptake behavior can be utilized for the treatment of cocaine-related substance use disorder. Based on this hypothesis, we designed KM822, an allosteric modulator of DAT using the hybrid structure-based screening method and demonstrated that KM822 can modulate the effects of cocaine on DAT without affecting dopamine uptake rates (Aggarwal et al., 2019). KM822 binds to a new allosteric site lined by residues Trp84, Tyr88, Phe155, Tyr156, Phe472, and His477 and hydrophilic residues Arg85, Lys384, Asp385, Thr473, and Asp476. This allosteric site is different from the new allosteric site for cocaine discovered by molecular docking and MD simulation (Xu and Chen, 2020). These findings regarding binding to the monomeric state of DAT raise the question as to whether the binding site is conserved in the oligomeric forms of DAT. It is clear that ligands like AIM-100 and methamphetamine are capable of inducing the oligomerization of DAT (Baucum et al., 2004; Sorkina et al., 2021). To understand whether they bind to the same site in oligomers as they do in the monomer, Cheng et al. (2019) did an extensive study on the trimerization of DAT induced by AIM-100 combining MD simulations, site-directed mutagenesis, and live-cell imaging. Their results show that the cavity at the trimeric interface forming the binding site for AIM-100 in oligomers was significantly different from the S2 binding site in the protomer (Cheng et al., 2019). These findings need validation using cryoEM or other methods to further understand the molecular architecture of DAT oligomers.

## 7 Selectivity of DAT compared to SERT and NET

DAT, SERT, and NET show an elevated degree of structural and sequence similarity with ∼40% sequence identity in humans making design of transporter-targeted drugs challenging. It can be assumed that the selectivity of these transporters is dictated by the type of residues that are present on the binding sites; however, the conformational changes of the transporters during the transport cycle (Figure 2) can largely alter the structure of the binding sites complicating the rationale for the observed selectivity for known inhibitors. As discussed, these transporters consist of well-defined ligand binding sites S1 and S2 along with other allosteric binding sites which poses significant challenges to design selective and potent drugs to target specific transporters.

Due to the limited knowledge in the structural factors that dictate selectivity, past discoveries of selective inhibitors relied on hit-and-trial or structure-activity relationship (SAR) methods. Tatsumi et al. (1997) determined the selectivity of the 37 different antidepressants for human SERT, NET, and DAT using radioligand binding assays. It is interesting that all tricyclic and amino tricyclic antidepressants are SERT selective whereas all secondary amine tricyclic antidepressants are selective for NET over SERT. Rational design of selective inhibitors has become more prominent over the last decade due to the advent of computational tools and the availability of structural information. Santra *et al.* synthesized several potent pyran-based inhibitors that are selective for DAT-NET or SERT or equally inhibitory towards all three (Gopishetty et al., 2011; Santra et al., 2012). Computational docking studies indicate that the stereochemistry of the-OH group at the pyran ring plays a critical role in the binding of these inhibitors (Santra et al., 2021). The-OH group facilitates a H-bond interaction with the Asp residue (Asp79 in DAT, Asp473 in NET, and Asp98 in SERT). The-OH and-OCH3 groups in the amino-benzyl substituent group are important for the selectivity for DAT and NET over SERT.

It can be assumed that the non-conserved residues at the S1 and S2 sites along with the EL4 which acts as the gatekeeper for the inhibitor entry to the transporter dictate the selectivity of the ligands. Andersen et al. conducted a systematic mutational analysis of the non-conserved residues at the binding sites S1 and S2, and EL4, to investigate their effect on the selectivity of substrates and inhibitors to these transporters (Andersen et al., 2015; Andersen et al., 2016). In the case of hNET but not hDAT and hSERT, the non-conserved residues in the S1 site primarily control the selectivity of the ligands indicating that selectivity of these transporters is more complex than initially thought. Koldsø et al. (2013) conducted extensive modeling and docking studies and predicted that the primary binding sites of these transporters have some specific hydrophilic residues which dictate the selectivity of these transporters. The authors further show that the extra OH group in norepinephrine does not contribute significantly to the binding. Recently, Ortore et al. (2020) performed an extensive computational docking and radioligand binding study on three human monoamine transporters and docking of 19 commonly known inhibitors revealed non-conserved residues as key elements for transporter selectivity. For example, Trp84, Arg85, Asp476, Pro387, and Phe472 dictate the hDAT selectivity and Phe431 and Ile172 are critical for hSERT selectivity. More recently, it was shown that EL2 and EL4 limit the access of the inhibitors to their binding site in SERT and act as the selectivity filter (Esendir et al., 2021). Sweeney *et al.* designed various chimeric structures to demonstrate the importance of C-and N-terminal regions in the substrate and common inhibitor affinities (Sweeney et al., 2017). Their result reveals that C-and N-terminal regions are also important for substrate and common inhibitor affinities for hDAT.

An interesting question is how these transporters are selective toward their corresponding neurotransmitter substrates compared to other transporters? Larsen *et al.* indicated that human and rat SERTs are also excellent DA transporters, but the mechanism is quite different from their serotonin transport, perhaps involving a differential role for oligomers in the two processes (Larsen et al., 2011). It is interesting to note that DA transport has an even higher maximum velocity than that of serotonin transport, although the Km for DA uptake is higher than that for serotonin transport. Furthermore, the transport requires significantly higher concentration of Na^+^/Cl^−^ transport and was inhibited non-competitively in the presence of serotonin. A number of brain regions implicated in motivated behavior such as the prefrontal cortex, nucleus accumbens shell, and basolateral amygdala have low presence of DAT, and SERT and NET may become more prominent in volume transmission clearance of DA (Tatsumi et al., 1997). Besides the selectivity displayed towards various pharmacological drugs, the selectivity of DAT, SERT, and NET towards various abusive drugs like cocaine, amphetamine, and methamphetamine is also interesting. Previously, DAT was considered as the only target for psychostimulants; however, studies using DAT knockout mice have disproved this notion (Di Chiara and Imperato, 1988; Sora et al., 1998; Sora et al., 2001). This adds a further challenge to designing pharmacotherapeutic agents to treat psychostimulant addiction.

One very interesting development based on the differential selectivity of substrates for DAT, SERT, and NET is that of releasers with a mixed monoamine releasing profile (Chen and Reith, 2008). Just as certain DAT blockers have inhibitory effects at SERT and/or NET, a DA releaser can also exert a releasing effect on 5-HT and/or NE and increased 5-HT release *in vivo* blunts phenotypic DA-mediated behaviors (Baumann et al., 2011). When additional substitutions to a substrate drug makes it less transportable, a partial releaser can be the result (Rothman et al., 2012) and this can be observed in addition to a mixed (hybrid) profile where transmitter release at one monoamine transporter occurs but is combined with inhibition of transmitter uptake at another transporter (Blough et al., 2014). All of these complexities bestow properties to a drug making it part of the growing category of atypical monoamine transporter ligands (Chen and Reith, 2008).

## 8 DAT interactions with other proteins

DAT interacts with several other proteins to perform its function (Eriksen et al., 2010). For example, an *in vivo* study in rats revealed that the interaction between DA D2 receptor and DAT increases DAT localization to the plasma membrane and increases DA uptake activity and neurotransmission (Dickinson et al., 1999; Lee et al., 2007). Protein co-immunoprecipitation suggests that the IC3 loop of D2 receptor interacts directly with the NTD of DAT (Lee et al., 2007). It is also reported that pramipexole, a DA D3 receptor preferring agonist, can promote interactions of DAT with D2/D3 receptors and α-synuclein (Castro-Hernández et al., 2015). Interestingly, this physical interaction of DAT with D2/D3 receptors diminishes DAT reuptake activity contradicting previous results with D2 receptor (Lee et al., 2007). The direct interaction of DAT and other monoamine transporters with Syntaxin 1A is well studied (Quick, 2002; Sung et al., 2003). Syntaxin 1A interacts with the NTD of DAT and suppresses DA uptake as well as DAT phosphorylation (Lee et al., 2004; Carvelli et al., 2008; Cervinski et al., 2010). Further, direct interaction of DAT with Syntaxin 1A enhances amphetamine-induced DA efflux. Amphetamine facilitates the direct interaction of DAT with Syntaxin 1A which is dependent on the Ca2+/calmodulin-dependent protein kinase II (CaMKII) (Binda et al., 2008). Supporting the above findings, Syntaxin 1A knock out animals failed to show amphetamine-induced locomotor activity indicating amphetamine-induced DA efflux requires Syntaxin 1A (Lanzo et al., 2018). Rickhag et al. (2013) revealed that blocking the interaction of the CTD of DAT with other proteins like CaMKII diminishes amphetamine-induced DA efflux. Receptor for Activated C Kinase 1 (RACK1) is another adapter protein that directly interacts with DAT and regulates DAT activity which was validated by deletion of the DAT NTD (Franekova et al., 2008). PICK1 is a PDZ domain-containing protein that interacts with the LKV motif of the CTD of hDAT, although some studies have questioned the importance of the LKV motif (Torres et al., 2001; Bjerggaard et al., 2004). DAT uptake activity was increased by ∼2-fold when DAT was co-expressed with PICK1 in HEK-293 cells indicating positive modulation of DAT by the PICK1 protein (Torres et al., 2001). The interaction of DAT with PICK1 helps in ER export and surface expression of DAT (Bjerggaard et al., 2004). We will highlight DAT’s interactions with HIV associated protein Tat and α-synuclein, two highly disordered proteins.

### 8.1 Interactions of DAT with Tat

Transactivator of transcription (Tat) protein is a human immunodeficiency virus type 1 (HIV-1) secreted viral protein involved in viral replication (Feng and Holland, 1988). In patients treated with anti-retroviral therapies, low levels of Tat have been detected in the cerebrospinal fluid which has been correlated with HIV-associated neurocognitive disorders (HAND) (King et al., 2006; Bagashev and Sawaya, 2013; Zhu et al., 2018) which affects more than 50% of patients (Heaton et al., 2010). Several studies have suggested that Tat causes neurological disorders by interacting with monoamine transporters (DAT, serotonin, norepinephrine) as well as with N-methyl-D-Aspartate (NMDA) receptors in the central nervous system impairing their functional activity (Aksenova et al., 2006; Kumar et al., 2011; Purohit et al., 2011; Adeniran et al., 2021; Ayoub et al., 2023). Several experiments have confirmed that Tat protein interacts with DAT and decreases dopamine uptake activity leading to CNS neurotoxicity (Zhu et al., 2009). HAND is more severe in HIV-1 patients with cocaine use disorder due to the synergistic effect of Tat and cocaine in DAT function inhibition. Tat is a small non-structural polypeptide consisting of six domains and a NMR structure shows that other than a short helical peptide, the rest of the protein is highly disordered (Péloponèse et al., 2000; Campbell and Loret, 2009). The first region (residues 1–21) is a proline-rich domain, the second region (residues 22–37) is a cystine-rich domain, the third region (residues 38–48) is the hydrophobic-core domain, the fourth region (residues 49–59) is rich in basic residues (basic-domain), and the fifth region (60–72) is the glutamate-rich domain (Kuppuswamy et al., 1989). Region 1–72 is involved in viral transcription and is encoded by exon 1. The sixth region is the C-terminal region, is variable in length (73–86 or 73–101) depending on the HIV isolate and is encoded by exon 2. In an effort to identify interactions of Tat with DAT, protein-protein docking, and MD simulations were performed on Tat-DAT complex. The results from this study suggest that Tat allosterically modulates DAT and interacts with the outward-open conformation (Zhu et al., 2011; Midde et al., 2013; Yuan et al., 2015; Sun et al., 2019). DAT residues Tyr88, Lys92, His547, and Tyr470 are critical for binding Tat with Tyr470 forming a strong cation-π interaction with the Tat M1 residue. Similarly, His547 has hydrogen bonded interactions with Arg49 and Pro18 of Tat as confirmed by site-directed mutagenesis and DA uptake assays (Yuan et al., 2015). A contemporary site-directed mutation and computational modeling analysis indicates that Asp206 of hDAT is also a key player in Tat binding where Asp206Leu mutation inhibits Tat binding without altering the basal DA uptake (Quizon et al., 2021). Although this binding site of Tat is different from the orthosteric site at which DA binds, the allosteric site at which Tat is predicted to bind sterically obstructs the substrate entry pathway and hence may inhibit DAT function. Furthermore, since DAT cannot bind to Tat in other conformational states, it is probable that Tat binding would freeze the outward-open state and prevent DAT’s transitions to other states affecting its kinetics.

Developing an appropriate allosteric ligand that can bind to hDAT and inhibits Tat or cocaine binding to hDAT without altering DAT uptake activity is an active area of research. Ligands that can bind with the residues Tyr88 and/or Tyr470 and/or His547 without altering DA uptake activity of the hDAT would be ideal for inhibiting Tat with hDAT. A study by Sun *et al.* investigated the modulatory effects of two allosteric ligands SRI-20041 and SRI-30827 on cocaine- and Tat-induced inhibition of hDAT (Sun et al., 2017). Results indicate that the ligands were able to attenuate the inhibitory effect of cocaine and Tat on hDAT. In addition to the direct inhibition of DAT with Tat, there is also evidence that Tat may induce internalization of DAT and decrease its plasma membrane expression (Midde et al., 2012). Moreover, Tat inhibition of DAT is dependent on PKC-mediated phosphorylation (see Section 3).

### 8.2 Interactions of DAT with α-synuclein

α-synuclein is predominantly expressed by neurons in dynamic equilibrium between cytosolic and membrane-bound forms and plays a pivotal role in synaptic vesicle trafficking and neurotransmitter release. The multimeric membrane-bound form acts as the molecular chaperone assisting in the assembly of synaptic fusion proteins called the SNARE complex (Burré et al., 2010). A wide range of evidence suggested that α-synuclein is a curvature sensing and curvature generating protein and prefers to bind to membranes with high positive curvature (Nepal et al., 2018). α-synuclein contains 140 residues and consists of three domains: the N-terminal lipid-binding α-helix residues 1–60, amyloid-binding central domain (NAC) residues 61–95, and C-terminal acidic tail residues 96–140 (Wang C. et al., 2016). The NMR structure of human micelle-bound α-synuclein has been resolved; in an aqueous solution, α-synuclein adopts a random coil structure but it attains a secondary structure in membrane proximity (Ulmer et al., 2005). Co-immunoprecipitation studies suggest that α-synuclein directly interacts with DAT, modulating DAT’s uptake activity (Wersinger et al., 2003a). Single neuron confocal microscopy indicates that α-synuclein is distributed throughout the neuron but localizes to the plasma membrane in the presence of DAT (Butler et al., 2015). The non-amyloid part of α-synuclein interacts with the CTD of DAT (last 22 amino acid residues) to form a DAT/α-synuclein complex (Lee et al., 2001; Eriksen et al., 2010). There is conflicting evidence on whether α-synuclein positively or negatively modulates DA uptake activity of DAT. *In vitro* studies in ltk-mouse fibroblasts indicate that α-synuclein:DAT interactions promote an aberrant increase in DA uptake activity *via* clustering of DATs on the membrane inducing apoptosis (Lee et al., 2001). Supporting evidence from human neuronal cell lines reveal a 50% reduction in DAT activity upon 80% knockdown of α-synuclein (Fountaine and Wade-Martins, 2007). It was also shown that the knockdown of α-synuclein caused a decrease in surface expression of hDAT (Fountaine et al., 2008); conversely, Wersinger *et al.* (Wersinger et al., 2003a; Wersinger and Sidhu, 2003) demonstrated that α-synuclein decreases DAT uptake activity (35%–40%) in *vitro* studies with co-transfected cells. The authors pointed out that α-synuclein:DAT complexation does not alter DA affinity for DAT but decreases the velocity of DA uptake. Swant et al. (2011) also found that over-expression of α-synuclein decreases the uptake activity of DAT and depolarizes the cell from −36.5 mV to −20.3 mV. Decreased DA uptake by α-synuclein was also reported by Butler et al. (Butler et al., 2012; Butler et al., 2015). α-synuclein is linked to the pathogenesis of PD (Spillantini et al., 1998) and in this context it is of interest that deficient DA uptake has also been linked with early-onset PD in multiple studies by Kurian and others (Reith et al., 2022).

Although mutation of α-synuclein is not a requirement for neurotoxicity (Moussa et al., 2004), Ala30Pro and Ala53Thr mutants are commonly detected in early-onset PD (Petrucelli et al., 2008). Expression of either of these mutants with hDAT in HEK-293 cells induces substantial ATP loss when exposed to 6-hydroxydopamine; however, this loss is absent without hDAT indicating hDAT: α-synuclein interactions are required for cellular toxicity (Lehmensiek et al., 2006). A set of separate studies show that cytotoxicity of Ala30Pro and Ala53Thr mutants is debated (Wersinger et al., 2003b; Moussa et al., 2004) and may be cell-type specific (Wersinger et al., 2004). Other functions of α-synuclein include modulation of DAT plasma membrane trafficking (Oaks et al., 2013) and clathrin-mediated endocytosis (Kisos et al., 2014).

Modulation of DAT activity by α-synuclein observed *in vitro* is not recapitulated *in vivo*. α-synuclein knockdown transgenic mice did not show any alteration of DAT levels in the striatum. Furthermore, α-synuclein null mice displayed remarkable resistance to neurotoxin MPTP-induced degeneration of DA neurons (Dauer et al., 2002). Simultaneous alpha- and gamma-synuclein knocked down transgenic mice showed a high level of extracellular DA due to higher DA release (Senior et al., 2008). Triple-synuclein-null mice revealed a substantial decrease in synaptic DA neurotransmission in dorsal striatum without alterations in the number of dopaminergic neurons (Anwar et al., 2011). SYN120—a human α-synuclein transgenic mice showed DAT/α-synuclein complexes that were significantly redistributed in the striatum and substantia nigra with higher expression of striatal DAT than wild type (Bellucci et al., 2011). However Ala53Thr mutant α-synuclein expressing transgenic mice did not show any change in DAT levels in the striatum compared to wild type (Oaks et al., 2013).

## 9 Conclusion

The crystallization of the related bacterial protein LeuT and dDAT has greatly increased our knowledge of hDAT’s structure and subsequently our understanding of the binding sites and conformational changes of DAT during substrate transport. The availability of these structure has accelerated the development of new DAT ligands with therapeutic potential, especially atypical drugs such as the allosteric modulator class. This structural knowledge has revealed post-translational modification sites of DAT; however, there is a lack of consensus regarding the functional consequences of DAT phosphorylation. The dynamics of DAT oligomerization and the role of cholesterol during these conformational transitions are poorly understood and stymied by the use of systems that may not fully reflect the native environment. Despite our current knowledge there is still a gap in our understanding of the conformational states of hDAT during the translocation cycle, exacerbated by the lack of an hDAT crystal or cryo-EM structure. Nevertheless, the available structural and functional knowledge of DAT has already spurred the development of drugs that regulate DAT in ways not understood before, allowing optimism regarding our capability of generating novel treatments. In particular, our existing knowledge of DAT’s interaction with other proteins including HIV-1 Tat and α-synuclein could potentially drive development of other novel disease-specific therapeutics.
